# Neuron-immune mechanisms contribute to pain in early stages of arthritis

**DOI:** 10.1186/s12974-016-0556-0

**Published:** 2016-04-29

**Authors:** Francisco R. Nieto, Anna K. Clark, John Grist, Gareth J. Hathway, Victoria Chapman, Marzia Malcangio

**Affiliations:** Wolfson Centre for Age-Related Diseases, King’s College London, Wolfson Wing, Hodgkin Building, Guy’s Campus, London, UK; Arthritis Research UK Pain Centre, School of Life Sciences, University of Nottingham, Nottingham, UK

**Keywords:** Collagen-induced arthritis, Pain, EMG, Microglia, IL-1β, Cathepsin S, P2X7 receptor

## Abstract

**Background:**

Rheumatoid arthritis (RA) patients frequently show weak correlations between the magnitude of pain and inflammation suggesting that mechanisms other than overt peripheral inflammation contribute to pain in RA. We assessed changes in microglial reactivity and spinal excitability and their contribution to pain-like behaviour in the early stages of collagen-induced arthritis (CIA) model.

**Methods:**

Mechanically evoked hypersensitivity, spinal nociceptive withdrawal reflexes (NWRs) and hind paw swelling were evaluated in female Lewis rats before and until 13 days following collagen immunization. In the spinal dorsal horn, microgliosis was assayed using immunohistochemistry (Iba-1/p-p38) and cyto(chemo)kine levels in the cerebrospinal fluid (CSF). Intrathecal administration of microglia-targeting drugs A-438079 (P2X7 antagonist) and LHVS (cathepsin S inhibitor) were examined upon hypersensitivity, NWRs, microgliosis and cyto(chemo)kine levels in the early phase of CIA.

**Results:**

The early phase of CIA was associated with mechanical allodynia and exaggerated mechanically evoked spinal NWRs, evident before hind paw swelling, and exacerbated with the development of swelling. Concomitant with the development of hypersensitivity was the presence of reactive spinal microgliosis and an increase of IL-1β levels in CSF (just detectable in plasma). Prolonged intrathecal administration of microglial inhibitors attenuated the development of mechanical allodynia, reduced microgliosis and attenuated IL-1β increments. Acute spinal application of either microglial inhibitor significantly diminished the sensitization of the spinal NWRs.

**Conclusions:**

Mechanical hypersensitivity in the early phase of CIA is associated with central sensitization that is dependent upon microglial-mediated release of IL-1β in the spinal cord. Blockade of these spinal events may provide pain relief in RA patients.

## Background

Rheumatoid arthritis (RA) is a frequent autoimmune disease characterized by synovial inflammation and joint damage [1]. Pain is the most dominant and disabling symptom reported by patients in both preclinical and clinical phases of this disease [2]. For instance, in the preclinical phase, symmetrical joint pain and morning stiffness correlate significantly with progression to arthritis [3]. Clinically induced remission in RA aims to control peripheral inflammatory processes in order to limit structural damage and functional impairment [4, 5]. However, RA patients may report pain before inflammation and pain may persist despite control of inflammation suggesting that mechanisms other than overt peripheral inflammation contribute to pain in RA. Consistent with this clinical observation, we and others have reported the presence of pain behaviours before the onset of clinical signs of RA as well as after resolution of joint inflammation in models of RA [6–11]. Thus, the clear disconnect between overt joint inflammation and pain symptoms presented by RA patients is replicated in the animal models.

For example, in the rat collagen-induced arthritis (CIA) model of RA, the onset of the clinical signs of arthritis is between 11 and 14 days after collagen immunization; however, pain-like behaviours are observed 7 days after immunization (early-stage CIA) and are then further exacerbated with increasing clinical scores (peak stage CIA) [9, 11]. CIA in rodents is a widely studied model of RA, largely on the basis of pathological similarities between the model and human disease; thus, rats display a severe polyarthritic phenotype consisting of swollen extremities, cartilage degradation, bone erosions and eventual loss of joint function, which is, in some aspects, similar to RA [12, 13]. CIA primarily affects ankle joints, with a significantly less proportion of animals with an involvement of knee joints [14], and based on macroscopic and microscopic patterns, CIA progression can be divided into three stages (always observed first in the hind paws): (1) preclinical (from collagen injection to clinically evident disease onset); (2) acute clinical (where hind paw swelling, body weight loss, inflammation and hind paw bone erosion were steadily progressing and macroscopic signs of CIA appeared on the fore paws); and (3) chronic clinical, where clinical (hind and fore paw swelling) and structural (inflammation and articular erosions in hind paws) evidence of joint involvement plateau [15].

In the early-stage CIA, there is a mild infiltration of inflammatory cells into the joint and significant upregulation in the expression of key receptors and channels in the cell bodies of nociceptive fibres innervating the ankle joints that are indicative of sensory hypersensitivity [11]. No swelling of the fore paws or knee joints is observed, just ankle joint inflammation that starts to spread down to the paw [11, 15, 16].

There is a growing consensus that in some RA patients persistent pain results from sensitization of the central pain pathways which is not correlated with synovial inflammation [17]. This concept is so well accepted that a prognostic tool that predicts the possible outcome of pain treatment with anti-inflammatory drugs has become available for rheumatologists [17]. Understanding the mechanism by which synovial inflammation induces central sensitisation is essential for effective pain management [2]. Like in other forms of chronic pain, central sensitization in RA is likely to arise from increased activation of primary sensory fibres (peripheral sensitization) [18]. For instance, nociceptor afferents innervating muscles or joints produce a longer-lasting central sensitization than those that innervate skin [18]. The same cytokines that drive early immune activation in preclinical stages of RA [19] sensitise primary afferent fibres in the joint [20]. In the dorsal horn of the spinal cord, increased excitation and reduced inhibition of pain signalling induces plastic changes in both neurons and non-neuronal cells [9, 11, 21]. In particular, spinal glial cells, especially microglia, contribute to chronic pain associated with arthritis by releasing proteases and cytokines that facilitate neuronal excitability [6–9, 22, 23]. In this scenario, peripheral inflammation in the joints is mirrored by an as-yet undefined central inflammation in the spinal cord. Intriguingly, existing clinical evidence indicate that pro-inflammatory cytokine levels, including IL-1β, are elevated in cerebrospinal fluid (CSF) of RA patients [24, 25].

In this study, we evaluated whether pain-like behaviour in early-stage CIA correlated with changes in microglial reactivity and spinal excitability in the spinal cord.

## Methods

### Animals

Experiments were performed in 152 female adult Lewis rats weighing 180–200 g (Charles River Laboratories, UK). Experimental study groups were randomized and blinded. All experiments were undertaken with approval of the UK Home Office and conformed to the ARRIVE Guidelines [26].

### Induction of arthritis

Induction of arthritis was performed as described previously [9, 11]. Briefly, bovine type II collagen (4 mg/ml; MD Bioproducts) was dissolved in acetic acid (0.1 M) and then emulsified with Freund’s complete adjuvant (CFA) 1 mg/ml (BD Biosciences). Rats were anaesthetized with isoflurane (Abbott) and injected intradermally at the base of the tail with 200 μl of collagen/CFA emulsion (400 μg of collagen per rat) or CFA emulsion (control rats).

### Macroscopic assessment of arthritis

Rats were scored on a scale of 0–3 per hind paw, 0–6 per rat [9, 11]. The emergence of ankle swelling, the earliest visible sign of arthritis, was scored as 1. Thereafter, footpad swelling occurred and was scored as 2. Subsequent swelling of one or more digits resulted in a score of 3. The thickness of each hind paw was measured using a thickness gauge (Mitutoyo) and expressed in millimetres. Body weight were monitored prior to immunization and then on throughout the disease process.

### Pain behaviour

Mechanical hypersensitivity of the hind paws was assessed as a measure of secondary hyperalgesia. Changes in hind paw mechanical withdrawal thresholds were assessed by applying a series of calibrated von Frey filaments (0.4–15.0 g, North Coast Medical) to the plantar surface of the hind paw according to the ‘up-down’ method [11]. On each day of testing, rats were habituated for 15 min in individual transparent plexiglass boxes with a wire mesh bottom, in a temperature-controlled room (22 °C). Calibrated von Frey filaments were applied to the plantar surface of the hind paw for 4–5 s or until the paw was withdrawn. Mechanical thresholds of the left and right paws were assessed alternately. Each test started with application of the 2-g filament. Once a withdrawal response to a von Frey hair was established, the paw was re-tested, starting with the filament below the one that produced a withdrawal, and subsequently with the remaining filaments in descending sequence until no withdrawal occurred and then ascending order until a response was observed once again. This up-down method was continued until the ‘*k*’ value could be calculated (between 4 and 9 applications of the von Frey hairs). From this, 50 % withdrawal thresholds were calculated.

### In vivo electrophysiology

The experimental setup for electromyographic (EMG) recordings have been previously described [27]. Briefly, naïve, CIA and control rats at 4, 7 and 13 days post-immunization were anaesthetized (2.5 % isoflurane) (Abbot) in oxygen, and an endotracheal cannula was inserted for controlled ventilation with a small animal ventilator (Harvard Apparatus). Rats were placed in a stereotaxic frame (Kopf Instruments). Body temperature was maintained at 37 ± 0.5 °C via a rectal probe coupled to a homeothermic heating blanket. Bipolar concentric needle EMG electrodes (Ainsworks) were placed through a small skin incision into the belly of the biceps femoris muscle of the right hind leg. Isoflurane anaesthesia was reduced to 1.5 % for 30–40 min prior to recording (at a level at which animals were moderately responsive to brushing of the cornea). The isoflurane concentration was maintained at the same level throughout the whole recording period. EMG (full-wave rectified) activity was recorded following sequential (lowest to highest) von Frey hair (15–180 g) stimulation of the plantar surface of the right foot. Raw EMG signals were conventionally amplified and displayed and fed to an analogue-to-digital converter for further analysis using a CED Micro1401 interface and Spike2 software (Cambridge Electronic Design). Each hair was applied three times, and the mean EMG response for each of the three presentations was calculated. Resting activity was subtracted from responses. Mechanical thresholds for each animal were determined as the lowest von Frey hair that produced an EMG response that was 10 % greater than the resting activity. A stimulus-response curve was plotted, and the area under the curve (AUC) was calculated to provide an overall measure of the spinal ‘reflex excitability’.

In experiments studying the effects of the spinally administered drugs, a small laminectomy [28] was performed to expose segments L4 and L5 of the spinal cord from CIA or control rats at day 7 post-immunization prior to insertion of the EMG electrode (see above). Following the laminectomy, isoflurane concentration was reduced to 1.5 % for 30–40 min prior to recording. Baseline EMG mechanical threshold and the baseline reflex excitability were established, and the effects of direct spinal administration of the P2X7 receptor antagonist A-438079 (50 μg/25 μl; Tocris Biosciences) or vehicle (saline) on mechanically evoked EMG responses (as described above) were recorded for 90 min. In a separate group of rats, morpholinurea-leucine-homophenylalaninevinyl phenyl sulfone (LHVS; 50 nmol/25 μl; NeoMPS Inc.), an irreversible, synthetic inhibitor of cathepsin S (CatS) or vehicle (20 % Cremophor EL/saline, Sigma-Aldrich), were applied to the spinal cord and effects on mechanically evoked EMG responses were recorded for 90 min. Doses of A-438079 and LHVS were based on previous studies ([29, 30], respectively).

### Intrathecal delivery of compounds in freely behaving rats

Under anaesthesia (a mixture medetomidine 0.25 mg/kg [Pfizer] and ketamine 60 mg/kg [Boehringer-Ingelheim]), a small laminectomy was performed at the sixth thoracic vertebra and a flexible cannula was inserted under the dura mater and glued in place, such that the tip is rested at the lumbar enlargement of the spinal cord. The opposite end of the cannula was placed subcutaneously, and an osmotic minipump (ALZET, Charles River Laboratories) was connected to the cannula [9, 11]. For the first pharmacological experiment, the P2X7 receptor antagonist A-438079 (50 μg/12 μL/day) or saline was delivered for 8 days (from 1 day before to 7 days post-immunization). For the second experiment, rats received the irreversible CatS inhibitor LHVS (30 nmol/12 μL/day) or vehicle (20 % Cremophor EL/saline) for 14 days beginning 1 day before and until day 13 post-immunization. Doses of A-438079 and LHVS were based on previous studies ([29, 30], respectively).

### Immunohistochemistry

Naïve rats, CIA and control rats on days 4, 7 and 13 post-immunization were anaesthetised (pentobarbital) and transcardially perfused with 0.9 % saline solution followed by 4 % paraformaldehyde with 1.5 % picric acid in 0.1 M phosphate buffer (pH 7.4). Lumbar spinal cords were dissected, post-fixed for 4 h in the perfusion fixative (4 °C), cryoprotected in 20 % sucrose in phosphate buffer (0.1 M, 4 °C) for 48 h and frozen in OCT embedding compound (VWR). Spinal cord (20 μm) sections were cryostat cut and thaw mounted onto Superfrost Plus Microscope Slides (VWR). Slides containing every sixth section of the lumbar (L4, L5) spinal cord were incubated overnight with rabbit anti-phospho-p38 mitogen-activated protein kinases (MAPK) (1:100; Cell Signaling) and then with the appropriate secondary biotinylated antibody for 90 min, followed by two stages of signal amplification with avidin-biotin complex (Vector Laboratories Inc.) and biotinyl tyramide (PerkinElmer) and finally visualized with ExtrAvidin-FITC as previously described [9]. Sections were then incubated overnight with the second primary antibody, rabbit anti-ionized calcium-binding adapter molecule 1 (Iba-1; 1:1000; Wako), followed by the appropriate secondary antibody (Alexa Fluor-546, Invitrogen). In separate experiments, slides of the lumbar spinal cord were incubated overnight with the primary antibody rabbit anti-glial fibrillary acidic protein (anti-GFAP; 1:1,000; DakoCytomation), followed by the appropriate secondary antibody (Alexa Fluor 488, Invitrogen). In control experiments, primary antibody was omitted whereby staining was completely abolished. All antibodies were prepared in PBS with 0.1 % Triton X-100. Slides were coverslipped with VECTASHIELD mounting medium with 2-(4-amidinophenyl)-1H-indole-6-carboxamidine (DAPI) (Vector), and images were captured using a Zeiss Axioplan-2 fluorescence microscope. Quantitative assessment of Iba-1 immunostaining in the spinal cord sections was performed by counting the positive profiles (Iba-1 indicative of microglial proliferation and p-p38 as an indication of microglia reactivity) within a fixed area of the superficial dorsal horn (boxes measuring 20^4^ μm^2^ were placed onto areas of the lateral, central and medial dorsal horns) (three sections/rat) as previously described [9, 11]. Quantitative assessment of GFAP immunostaining in the spinal cord sections was performed by measuring the intensity of the GFAP immunoreactivity within a fixed area of the superficial dorsal horn (boxes measuring 20^4^ μm^2^ were placed onto areas of the lateral, central and medial dorsal horns) (three sections/rat) as previously described [9].

### Cytokine and chemokine measurement assays

Cerebrospinal fluid (CSF) and blood samples were collected from naïve, CIA and control rats at 4, 7 and 13 days post-immunization. Following cardiac puncture under pentobarbital anaesthesia, plasma aliquots were obtained from blood samples. Then, the skin covering the occipital bone and the cervical dorsum was incised, and the occipital bone and upper cervical vertebral arc were exposed. The atlanto-occipital membrane was identified and carefully cleared of surrounding tissues. The needle of a 29-gauge insulin syringe was inserted horizontally through the lateral atlanto-occipital membrane, and 50–100 μl of CSF was withdrawn and immediately flash frozen in liquid nitrogen [31].

IL-1β, IL-6, TNF-α, IL-10 and MCP-1 concentrations in plasma and CSF samples were measured using a Luminex-based multiplex immunoassay following the manufacturer’s instructions (eBioscience Inc.). Fractalkine (FKN) (CX3CL1) levels in CSF were quantified with enzyme-linked immunosorbent assay (ELISA) following the manufacturer’s instructions (RayBiotech Inc.).

### Data analysis

Differences between values in the behavioural tests were analysed with two-way repeated measures analysis of variance (RM-ANOVA) followed by Tukey’s test. EMG, immunohistochemistry, multiplex immunoassay and ELISA data were analysed by two-way ANOVA followed by Tukey’s test, by one-way ANOVA followed by Tukey’s test, or *t* test, respectively, as appropriate. Analyses of correlations were performed with the mean values of each parameter, and the number of animals were not the same in the different parameters analysed as specified in the figure legends. Analyses of correlations were determined with a Pearson correlation test. Data are shown as mean ± SEM. The differences between means were considered statistically significant when *P* < 0.05.

## Results

### CIA is associated with a reactive spinal microgliosis that correlates with the time course of mechanical hypersensitivity (allodynia)

We previously reported that hind paw swelling, clinical signs and mechanical hypersensitivity in CIA rats at the peak of disease (day 18 post-immunization) are associated with significant microglial reactivity in the dorsal horn of the spinal cord which contributes to pain hypersensitivity, but not to joint swelling and clinical scores [9].

**Fig. 1:**
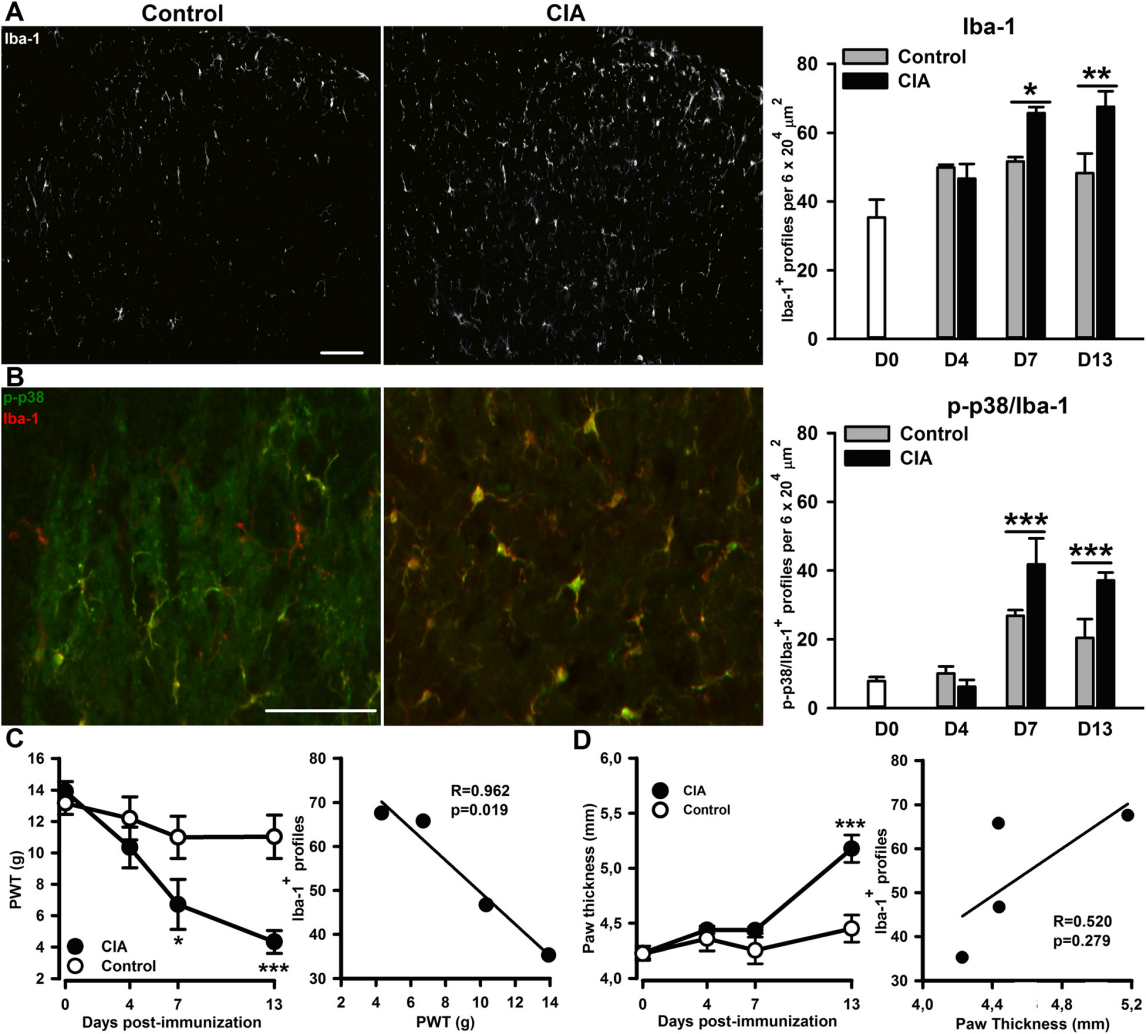
Enhanced microglial response in the spinal cord dorsal horn of collagen-immunised (CI) rats. Ionized calcium-binding adapter molecule 1 (*Iba-1*) immunoreactivity in the lumbar dorsal horn of control and CI rats on day 13 post-injections (**a**). Co-localization of Iba-1 (*red*) and phospho-p38 (*green*) immunoreactivity (*yellow*) in the dorsal horn (**b**). Quantification of Iba-1 (**a**) and phospho-p38/Iba-1 (**b**) in the dorsal horn. Scale bars = 100 μm. In **a** and **b**, values are the mean ± SEM of 5–4 rats per group. ***p < 0.001, **p < 0.01, *p < 0.05 versus controls; two-way ANOVA, post hoc Tukey’s test. Time course of mechanical hypersensitivity associated to collagen immunization (panel **c** *left*), which significantly correlates with the time course of the microglial response (panel **c** *right*). Time course of hind paw swelling associated to collagen immunization (panel **d** *left*), which does not correlate with the time course of the microglial response (panel **d** *right*). In **c** and **d**, values are the mean ± SEM of 8 rats per group: ***p < 0.001, *p < 0.05 versus controls; two-way RM-ANOVA, post hoc Tukey’s test. In correlations studies in **c** and **d**, data were analysed with a Pearson correlation

**Fig. 2:**
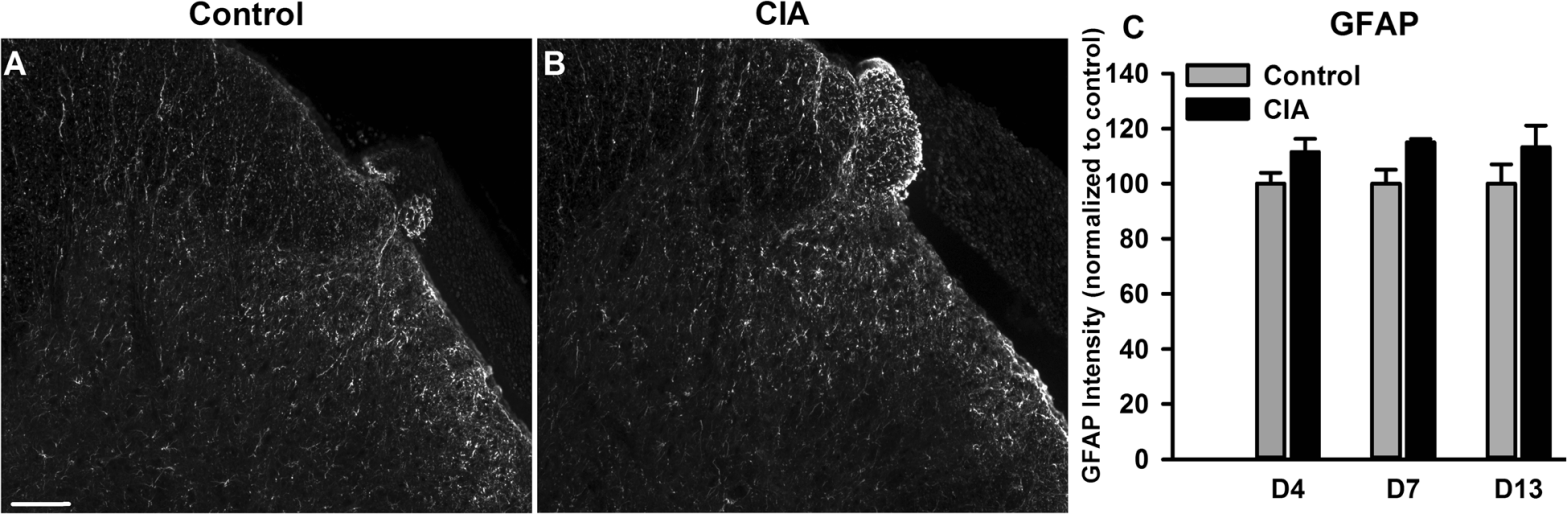
Collagen-induced arthritis (*CIA*) is not associated with a significant astrogliosis in the dorsal horn of the spinal cord. Glial fibrillary acidic protein (*GFAP*) immunoreactivity in the dorsal horn of control (**a**) and CIA (**b**) rats on day 13 post-injection. **c** Quantification of GFAP intensity in the dorsal horn of CIA and control rats. Scale bar = 100 μm. In **c**, values are the mean ± SEM of 4 (days 4, 7 and 13) rats per group. No statistically significant differences (two-way ANOVA)

At 7 and 13 days following collagen immunization, Iba-1^+^ profiles in the lumbar dorsal horn were significantly increased compared to control rats [CIA *F*(1,26) = 6.62, *P* = 0.016; Fig. 1a]; in addition, there was a significant increase in Iba-1^+^ phosphorylated p38 MAPK coexpression [CIA *F*(1,26) = 14.7, *P* < 0.001; Fig. 1b)]. We did not find evidence of microgliosis in the ventral horn of the spinal cord of CIA rats (data not shown). The number of Iba-1^+^ profiles in the dorsal horn correlated significantly with mechanical hind paw withdrawal thresholds (Fig. 1c), but not paw swelling (Fig. 1d). In fact, the onset of mechanical hypersensitivity and microgliosis occurred on day 7 after collagen immunization in the absence of noticeable paw swelling (Fig. 1c, d). In contrast to spinal microgliosis, GFAP immunoreactivity, a marker of astrocytes, in the dorsal horn of the spinal cord in CIA rats was not different to control rats throughout the period of study (Fig. 2).

### CIA produces an exaggerated mechanically evoked spinal nociceptive withdrawal reflexes (NWRs) with a time course similar to that of mechanical allodynia

**Fig. 3:**
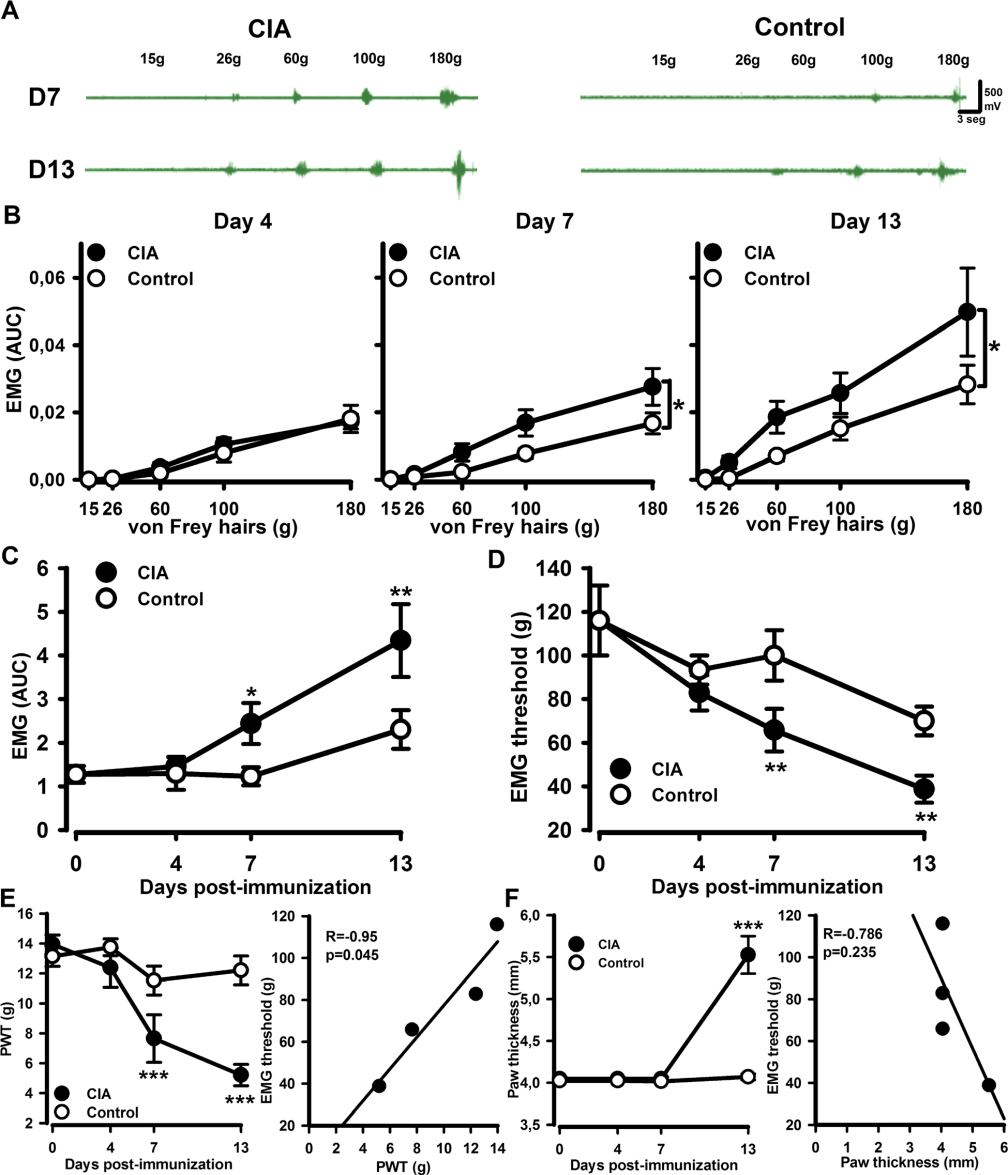
Enhanced mechanically evoked spinal nociceptive withdrawal reflexes (NWRs) in CIA rats. The plantar surface of hind paws were mechanically stimulated with von Frey hairs, and evoked biceps femoris (BF) EMG responses were recorded. **a** Representative raw EMG traces recordings BF muscle in CIA and control rats stimulated with different von Frey hairs. **b** Stimulus-response curve of EMG response versus mechanical stimulus intensity at different time points post CIA immunization. **c** AUC analysis of the time course of the overall reflex response to von Frey hairs of increasing intensity in control and CIA rats (**d**). Time course of the BF mechanical thresholds in control and CIA rats. Time course of CIA-associated mechanical hypersensitivity (panel **e** *left*) significantly correlated with the time course of the change in BF mechanical thresholds (panel **e** *right*). Time course of CIA-induced hind paw swelling (panel **e** *left*) did not correlate with the time course of the BF mechanical thresholds (panel **e** *right*). Values are the mean ± SEM of 6 (day 0), 7 (day 4), 9 (day 7) and 8 (day 13) rats per group. ***p < 0.001, **p < 0.01, *p < 0.05 versus controls, two-way RM-ANOVA, post hoc Tukey’s test. In **b**, statistical analysis performed with AUC analysis and *t* test. In correlations studies in **e** and **f**, data were analysed with a Pearson correlation

In order to evaluate whether pain hypersensitivity in early-stage CIA was associated with changes in spinal excitability, EMG responses were recorded in anesthetised rats. Mechanical stimulation of hind paw resulted in a significantly larger bicep femoris muscle EMG response in CIA rats, compared to control groups at days 7 and 13 following collagen immunization (Fig. 3a–c) [CIA *F*(1,49) = 6.27, *P* = 0.016; Fig. 2c]. In addition, mechanical thresholds needed to evoke reflex responses were significantly lower in the CIA rats, compared to control groups [CIA *F*(1,49) = 6.78, *P* = 0.012; Fig. 3d)]. The decrease in EMG mechanical thresholds correlated significantly with decreased freely behaving paw withdrawal thresholds (Fig. 3e), but not with hind paw swelling (Fig. 3f).

### IL-1β levels increase on days 7 and 13 in CSF whereas they are just detectable in plasma of CIA rats

**Fig. 4:**
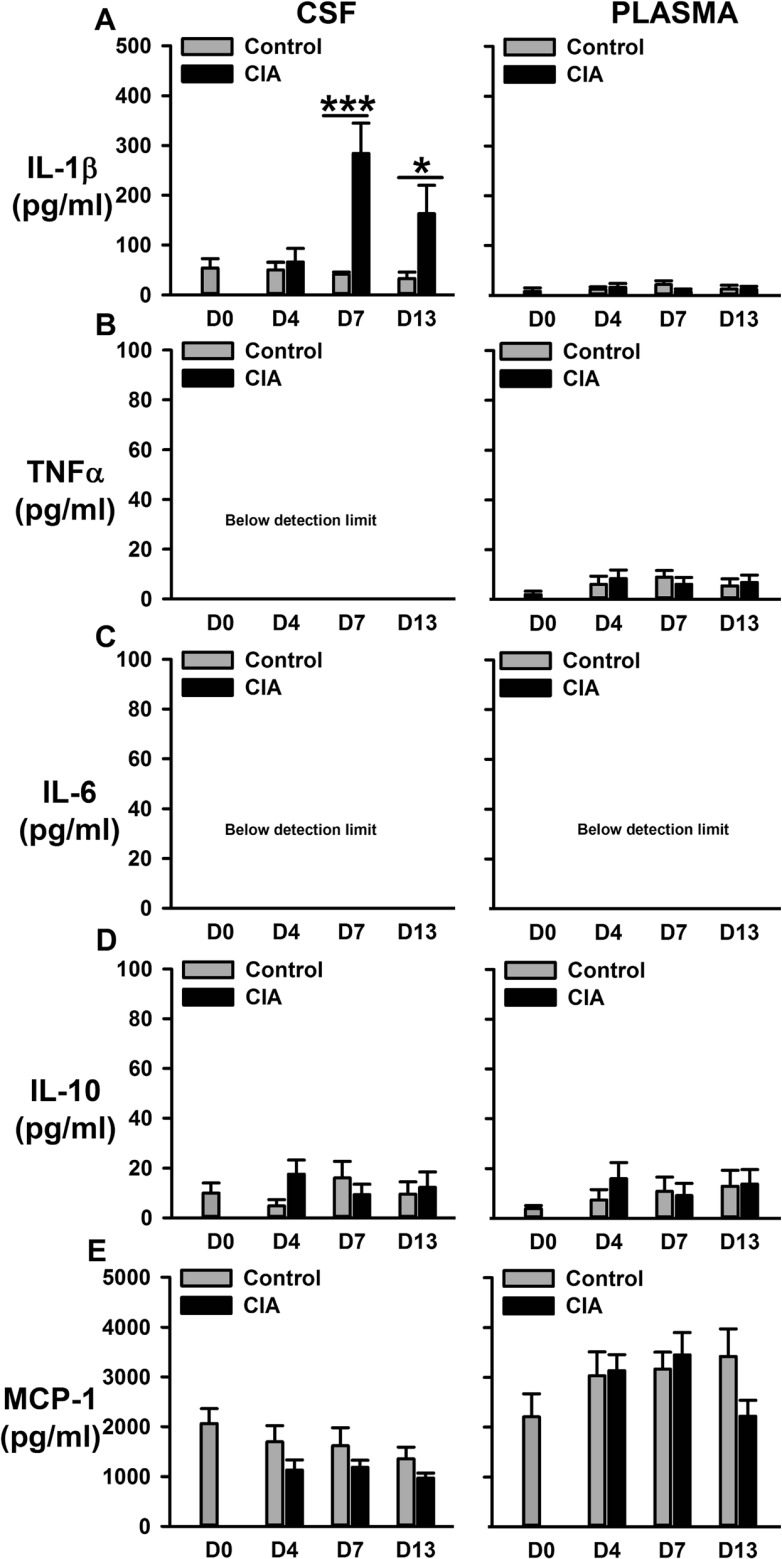
Levels of Il-1β (**a**), TNF-α (**b**), IL-6 (**c**), IL-10 (**d**) and MCP-1 (**e**) in CSF and plasma from CIA and control rats measured with multiplex immunoassay. Values are the mean ± SEM of 4–7 rats per group. ***p < 0.001, *p < 0.05 versus controls; two-way ANOVA, post hoc Tukey’s test

Next, we tested the hypothesis that reactive microglia contribute to neuronal sensitization in the spinal cord by releasing pro-nociceptive mediators. To achieve this, we determined cyto(chemo)kine levels in the CSF and plasma of rats in the early stages of CIA. Plasma cyto(chemo)kine concentrations (IL-1β, TNFα, IL-6, IL-10, MCP-1; Fig. 4a–e) in CIA rats remained stable and were not significantly different to those in control rats on days 4, 7 and 13 post-immunization; plasma IL-6 concentrations were below the detection limit of the assay that we employed (Fig. 4c). In contrast, IL-1β levels in the CSF were significantly increased on days 7 and 13 after collagen immunization, compared to controls [CIA *F*(1,32) = 7.16, *P* = 0.012; Fig. 4a]. CSF TNF-α (Fig. 4b) and IL-6 (Fig. 4c) levels were below detection limits, and IL-10 (Fig.4d) and MCP-1 (Fig. 4e) levels did not change significantly in CIA rats.

### Inhibition of spinal cathepsin S or P2X7 receptor prevents mechanical hypersensitivity, microglial reactivity and IL-1β increase associated to the early stages of CIA

**Fig. 5:**
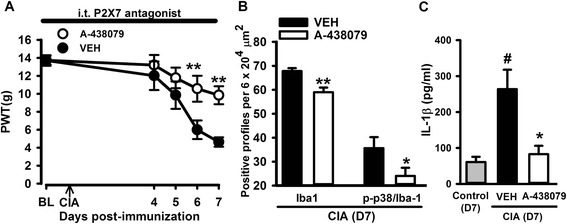
Spinal inhibition of P2X7 receptors during the early phase of CIA attenuated mechanical hypersensitivity, spinal microgliosis and increases in the levels of the pro-inflammatory cytokine IL1-β in CSF. Intrathecal (*i.t.*) administration of the P2X7 antagonist (A-438079, 50 μg/day) from 1 day before immunization until day 7 post-immunization significantly prevented mechanical hypersensitivity in CIA rats (**a**), reduced spinal microgliosis (**b**) and the increased levels of IL-1β in CIA rats (**c**). Values are mean ± SEM of 8 rats per group. ***p < 0.001, **p < 0.01, *p < 0.05 compared to vehicle group; #*p* < 0.05 compared to control group (**a** two-way RM ANOVA, post hoc Tukey’s test; **b** *t* test; **c** one-way ANOVA, post hoc Tukey’s test)

In the spinal cord, microglia release IL-1β following activation of the P2X7 receptor and extracellular IL-1β exerts pro-nociceptive effects [29, 32]. Microglial P2X7 receptors also mediate the release of the lysosomal protease CatS [33] which is pro-nociceptive in the dorsal horn via cleavage of the neuronal fractalkine into a soluble chemokine domain that activates microglial CX3CR1 receptors [31]. In order to examine whether reactive microglia played a role in central mechanisms of early-stage CIA pain hypersensitivity, we evaluated whether continuous intrathecal administration of a P2X7 receptor antagonist or a CatS inhibitor in the lumbar spinal cord for several days after collagen immunization prevented (i) IL-1β concentration increases in CSF, (ii) microglial reactivity in the spinal cord and (iii) hind paw mechanical hypersensitivity. Continuous intrathecal administration of A-438079 (P2X7 receptor antagonist) for 8 days significantly reduced the magnitude of mechanical allodynia compared to vehicle administration in CIA rats [A-438079 *F*(1,60) = 4.50, *P* = 0.044; Fig. 5a], attenuated spinal cord microgliosis and microglial reactivity (Fig. 5b) and prevented the increase of IL-1β which were all detected in the vehicle group [A-438079 *F*(2,23) = 5.51, *P* = 0.011; Fig. 5c)].

**Fig. 6:**
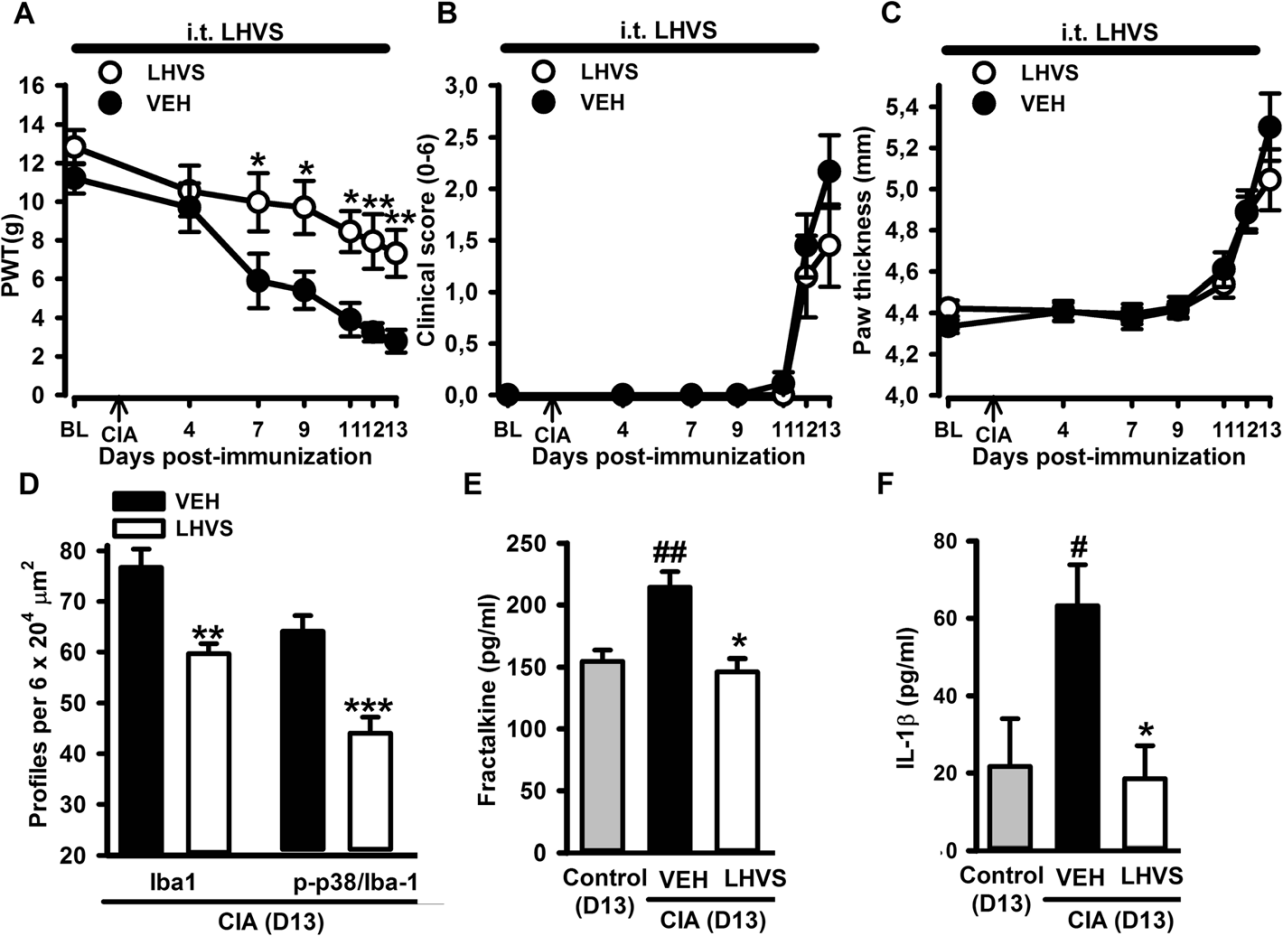
Spinal inhibition of cathepsin S (CatS) in the early phase of CIA reduced mechanical hypersensitivity, microgliosis and the increased levels of pro-inflammatory cyto(chemo)kines in CSF, without affecting paw swelling. Intrathecal (*i.t.*) administration of the CatS inhibitor (LHVS, 30 nmol/day) from 1 day before until day 13 post-immunization significantly prevented mechanical hypersensitivity in CIA rats (**a**) and did not modify the clinical score (**b**) or paw swelling (**c**). LHVS reduced spinal microgliosis (Iba-1/p-p38 (**d**) and the increased levels of fractalkine (**e**) and IL-1β (**f**) associated with CIA. Values are the mean ± SEM of 10 rats/group (**a**–**c**) and 4–6 rats/group (**d**–**f**). ***p < 0.001, **p < 0.01, *p < 0.05 compared to vehicle group; #*p* < 0.05 compared to control group (**a**–**c** two-way RM ANOVA post hoc Tukey’s test; **d** *t* test; **e**, **f** one-way ANOVA post hoc Tukey’s test)

Similarly, intrathecal administration of LHVS (CatS inhibitor) resulted in less hind paw mechanical hypersensitivity compared to vehicle administration in CIA rats [LHVS *F*(1,108) = 7.99, *P* = 0.011; Fig. 6a]. However, LHVS did not alter the onset of clinical scores (Fig. 6b) and paw swelling (Fig. 6c) which occurred 11 and 13 days post-immunization similar to vehicle-treated rats. LHVS administration also reduced microgliosis and microglial reactivity in the dorsal horn of the spinal cord in CIA rats, compared to vehicle (Fig. 6d). In addition, IL-1β and FKN levels in the CSF remained at basal control levels in the LHVS-treated group whilst they were significantly increased in vehicle-treated group of collagen-immunised rats [one-way ANOVA, *F*(2,24) = 5.82, *P* = 0.009, Fig. 6e; *F*(2,11) = 10.81, *P* = 0.003, Fig. 6f, respectively)].

### Inhibition of spinal cathepsin S or P2X7 receptor both reversed enhanced EMG activity associated to CIA

**Fig. 7:**
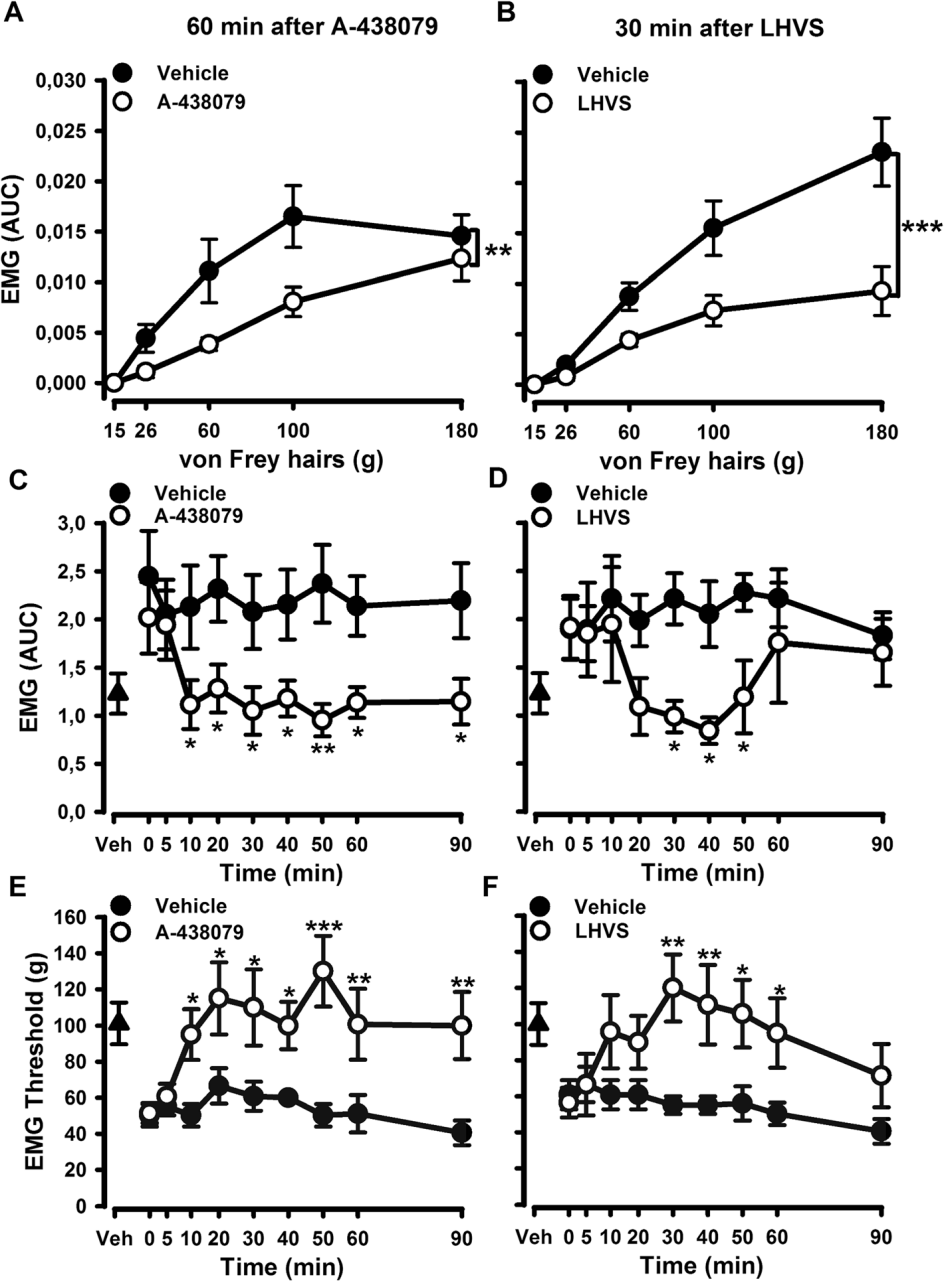
Acute spinal blockade of P2X7 receptors (A-437089) or inhibition of cathepsin S (CatS) (LHVS) attenuated the enhanced mechanically evoked biceps femoris (BF) EMG responses in CIA rats at day 7. Representative mechanical stimulus-EMG response curve at 60 and 30 min after spinal treatment with A-437089 (**a**) or LHVS (**b**) in CIA rats. Time courses of the effects of a single spinal dose of the A-438079 (50 μg per rat) or saline on the magnitude of evoked BF EMG responses (**c**) or BF mechanical thresholds (**e**) in CIA rats 7 days post CIA immunization. Time courses of the effects of a single spinal administration of LHVS (50 nmol per rat) or vehicle (20 % Cremophor EL/saline) on the magnitude of evoked BF EMG responses (**d**) or BF mechanical thresholds (**f**) in CIA rats 7 days post-immunization. Values are mean ± SEM of 7–8 rats per group. ***p < 0.001, **p < 0.01, *p < 0.05 compared to control group (two-way RM ANOVA, post hoc Tukey’s test). In **a** and **b**, statistical analysis performed with AUC analysis and *t* test

In the final set of experiments, we evaluated whether spinal application of A-438079 and LHVS altered spinal excitability as measured by the EMG response in the CIA rats. A-438079 significantly inhibited the enhanced mechanically evoked spinal nociceptive EMG activity in CIA rats from 10 to 90 min after spinal application [Fig. 7a, c, e; Fig. 7c A-438079 *F*(1,104) = 6.64, *P* = 0.023; Fig. 6e A-438079 *F*(1,104) = 12.82, *P* = 0.003)] whilst LHVS was significantly effective from 30 to 50–60 min after spinal application [Fig. 7b, d, e; Fig. 7d*F*(1,104) = 2.25, *P* = 0.157; Fig. 7e LHVS *F*(1,104) = 4.73, *P* = 0.049]. Altogether, these data indicate that in early CIA, mechanical hypersensitivity is associated with microglial reactivity and spinal excitability that are attenuated by inhibition of P2X7 receptor and CatS enzyme in microglia.

## Discussion

In this study, we show that the early phase of the CIA model in the rat is associated with mechanical allodynia and spinal hyperexcitability in parallel with enhanced microgliosis response and an increase of IL-1β levels in CSF, prior to clinical signs of CIA being evident. With the establishment of hind paw swelling, mechanical allodynia and spinal hyperexcitability intensify whereas spinal microgliosis and IL-1β remain at similar level. These central changes are probably mediated by microglia-driven mechanisms in which the P2X7-CatS/CX3CR1 pathway in the spinal cord exerts a central role. In fact, the inhibition of this pathway attenuated both mechanical allodynia and the hyperexcitability of the spinal cord without affecting the progression of the inflammation of the hind paws induced by CIA. Thus, these data suggest that mechanical hypersensitivity linked to CIA is likely mediated by a sensitization of spinal nociceptive networks, like other forms of chronic pain [34].

Pain reported by people suffering with RA arises from multiple mechanisms and are dependent not only upon peripheral inflammation with patients often presenting symptoms that are typical of an involvement of the central nervous system (CNS) [2]. Thus, people with RA present reductions in mechanical and thermal pain thresholds, not only over inflamed joints but also at non-inflamed regions adjacent to or even remote from the inflamed joints, and RA patients continue to have widespread pain, despite low levels of inflammation [4, 35]. In the present study, we also observed a poor correlation between pain and inflammation, since CIA rats develop a robust mechanical allodynia before the onset of hind paw swelling, which is in agreement with previous studies by our group [9, 11] and with other studies in mice [7, 10].

Here, we demonstrated that spinal nociceptive reflex pathways are sensitized as mechanically evoked EMG responses increase in rats immunised with collagen before the development of hind paw swelling with a further increase with the progress of the inflammation. The study of reflexes in humans and animal models is a neurophysiological tool used to measure changes in the excitability of spinal pain networks and its modulation during chronic pain [36–38]. To our knowledge, this is the first report which studies electrophysiological properties in a model of RA showing spinal hyperexcitability in parallel with the development of mechanical allodynia and spinal microgliosis, supporting the presence of central sensitization in the rat CIA model.

Microglia activation in the spinal cord is known to be a critical component of chronic pain conditions [39]. We and others previously described an enhanced microglia response associated with chronic pain in different RA models, focusing on later phases, and associated with the peak of inflammation [7–9], or when the inflammatory activity had stopped [6–8]. Mild infiltration of inflammatory cells into the ankle joints at day 7 after collagen immunization leads to the activation of sensory neurons innervating the joint and adjacent areas [11]. Such sensitized sensory neurons release pro-nociceptive transmitters from their central terminals in the superficial dorsal horn of the spinal cord. Here, such transmitters promote activation of microglia with a further release of pro-inflammatory mediators that act to enhance neuronal central sensitization and induce persistent pain. In summary, the immunization with collagen can produce a peripheral sensitization (activation of sensory neurons) that leads to a central sensitization in the spinal cord and altogether contribute to the pain states associated to CIA.

In the present study, we focussed on the early phase of CIA and observed a significant microgliosis (increase in Iba-1 and phospho-p38 expression) which is evident in the dorsal horn of the spinal cord at 7 days from collagen immunization (1 week before the onset of clinical signs), with a time course similar to that of mechanical allodynia. An increase in activation (phosphorylation) of p38 in the spinal microglia as reported here has been shown previously to be involved in the development of neuropathic [40, 41] and inflammatory [42, 43] pain. The processes by which microglia mediate increases in neuronal sensitivity are thought to involve the synthesis of proinflammatory cytokines that are released by microglia either in response to inflammation, injury or following C-fibre strength activation of primary afferent fibres [44, 45]. Accordingly, we studied the protein levels of several cyto(chemo)kines and report an increase of IL-1β in the CSF of CIA rats. In agreement with our results, Lampa et al. [24] reported an increase in levels of IL-1β mRNA in the spinal cord tissue in the K/BxN serum transfer RA model. As cytokines can cross the blood-brain barrier [46], and increments of cytokines in blood have been reported in the CIA model [47, 48], the source of increased concentrations of IL-1β in the CSF following immunization with collagen could be in the blood and/or from changes in the release of these inflammatory mediators from CNS resident cells (e.g. microglia and astrocytes; see above). Our investigations show that at the early time points of the CIA model, plasma levels of cytokines did not change, with IL-1β levels being significantly lower than those found in the CSF at the same time point. Interestingly, it has been recently reported that RA patients have elevated CSF concentrations of IL-1β, which are higher than the corresponding serum concentration which mirrors our investigations [25]. IL-1β in the spinal cord contributes to inflammatory pain hypersensitivity as an inducer of spinal COX-2 upregulation [49], which itself has been described to be upregulated in the spinal cord of CIA mice [50]. These data suggest that IL-1β released from spinal microglia has a significant role in the mechanisms of chronic pain associated to CIA.

CIA results from the immunization of animals with type II collagen, which induces an autoimmune disease directed against the cartilage in the joints. We previously described that mechanical hypersensitivity in the early phase of CIA develops in parallel with a mild infiltration of inflammatory cells into the ankle joint and activation of the sensory neurons innervating the ankle joint and adjacent areas [11]. Such sensitized afferent fibres release from their central terminals a variety of neurotransmitters, including glutamate, substance P, CGRP and ATP into the superficial dorsal horn of the spinal cord which can lead to the activation of glial cells and the further release of pro-inflammatory mediators like cytokines that act to enhance neuronal central sensitization and induce persistent pain [51]. In these studies, we have found a clear spinal microgliosis, we did not find evidence for spinal reactive astrocytosis in the early phase of CIA, suggesting that the source of the striking upregulation of IL-1β in CSF is mediated by microglia-driven mechanisms. In the spinal cord, microglia can release IL-1β following activation of the P2X7 receptor [29] present mainly in microglia [52, 53]. Here, we demonstrated that the spinal P2X7 receptor inhibition prevented mechanical hypersensitivity in the early phase of CIA in parallel with an attenuation of microgliosis and the increase of IL-1β, suggesting that spinal microglial cells release IL-1β via a P2X7-mediated pathway. In addition, we have previously demonstrated that microglial P2X7 receptor activation leads to the release of the protease CatS concomitantly to IL-1β [33]. Extracellular CatS is pro-nociceptive mediating its effects via cleavage of the chemokine domain of neuronally expressed FKN. This soluble fraction then is able to diffuse and reach microglial CX3CR1 receptor and further activates these cells [30, 31]. In the present study, we observed a significant increase of FKN in the CSF of rat by day 13 after collagen immunization. The pharmacological blockade of spinal CatS not only reduced the chemokine FKN to control levels but also prevented the allodynia and spinal microglial activation and the increase of IL-1β in CSF in CIA rat. Therefore, like in other models of chronic pain, antagonism of these microglial targets (the P2X7 receptor and the protease CatS) in the spinal cord reduces pain behaviours [29–31, 52, 53], and our data suggest this action is mediated through a mechanism involving a reduction in the release of IL-1β in the spinal cord. Further investigation of the mechanisms of this regulation in the spinal cord at the protein or mRNA levels is warranted in future studies.

EMG studies in animals have been employed to study the modulation of spinal cord nociceptive reflexes by the systemic or central administration of drugs [54, 55]. Here, we demonstrated that the acute inhibition of the P2X7 receptor or the protease cathepsin S in the spinal cord reduced the enhanced EMG responses induced by the immunization with collagen, indicating that the inhibition of these microglia-driven mechanisms is enough to reduce the spinal nociceptive reflex facilitation during the CIA model.

## Conclusions

In summary, our studies strongly support the involvement of central sensitization processes in the mechanical hypersensitivity associated with the early phase of the rat CIA model. This central sensitization is dependent upon microglial mechanisms, probably through the release of IL-1β in the spinal cord, with a key role of the microglial P2X7 receptor and the protease CatS. Our data suggest that the inhibition of these microglial targets by CNS-penetrating drugs could represent a new therapeutic opportunity for the treatment of pain suffered for RA patient, especially in those without severe clinical signs of arthritis.

## Abbreviations

AUC: area under the curve
CFA: Freund’s complete adjuvant
CIA: collagen-induced arthritis
CNS: central nervous system
CSF: cerebrospinal fluid
EMG: electromyographic
GFAP: anti-glial fibrillary acidic protein
Iba-1: ionized calcium-binding adapter molecule 1
MAPK: mitogen-activated protein kinases
NWRs: nociceptive withdrawal reflexes
RA: rheumatoid arthritis
